# Monoclinic polymorph of 2-aza­niumylmethyl-1*H*-benzimidazol-3-ium dichloride monohydrate

**DOI:** 10.1107/S2414314625008685

**Published:** 2025-10-07

**Authors:** Manjula Devi Baskaran, Shanthini Jayaraman, Madhukar Hemamalini, Mark R. J. Elsegood, Venkatachalam Rajakannan, Savaridasson Jose Kavitha

**Affiliations:** a Department of Chemistry Mother Teresa Women’s University, Kodaikanal Tamil Nadu India; b Chemistry Department, Loughborough University, Loughborough, Leicestershire, LE11 3TU, United Kingdom; c Department of Crystallography and Biophysics University of Madras, Guindy Campus Chennai-600 025 Tamil Nadu India; University of Aberdeen, United Kingdom

**Keywords:** crystal structure, hydrogen bonding, polymorph

## Abstract

The title hydrated salt, C_8_H_11_N_3_^2+^·2Cl^−^·H_2_O, is a monoclinic polymorph of the previously reported triclinic form. The compound crystallizes in space group *P*2_1_/*c* with *Z*′ = 1. The crystal structure features N—H⋯O, N—H⋯Cl and O—H⋯Cl hydrogen bonds and aromatic π–π stacking inter­actions, forming a three-dimensional network.

## Abstract 2

The title hydrated salt, a new monoclinic polymorph, crystallizes in space group *P*2_1_/*c*. The components pack in sheets in the *ab* plane and these sheets are hydrogen bonded to their neighbours, generating a three-dimensional network.

## Graphical abstract

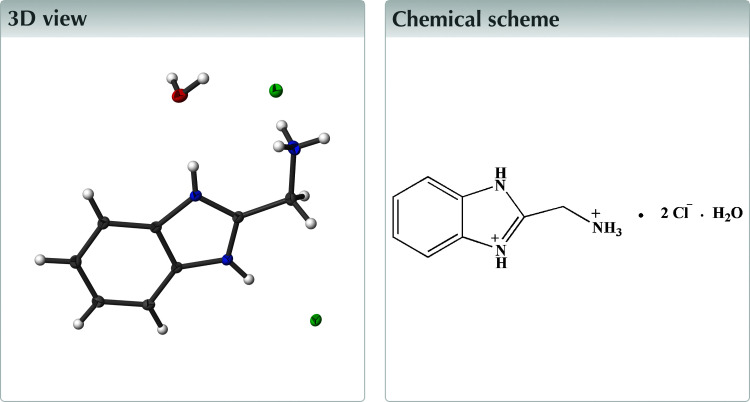

## Structure description

Benzimidazole-based systems have attracted attention as ligands for metal complexation owing to the desirable properties that are beneficial for biological applications such as anti-bacterial (Kankate *et al.*, 2019 ▸) and anti-hypertensive (Sharma *et al.*, 2013 ▸) effects. The materials applications of benzimidazole ligands include luminescent properties of their metal complexes, which can be applied in electroluminescent devices (Wu *et al.*, 2008 ▸). The larger conjugated π-system and the nitro­gen electron donor of the secondary amine group of the benzimidazole moiety play an important role in determining the properties of the complexes. As part of our work in this area, we now describe the synthesis and structure of the title benzimidazolium salt, C_8_H_11_N_3_^2+^·2Cl^−^·H_2_O.

A search of the Cambridge Structural Database (Version 6.00, update April 2025; Groom *et al.*, 2016 ▸) for the 2-ammonium­ylmethyl-1*H* -benzimidazol-3-ium (C_8_H_11_N_3_^2+^) dication, generated three hits: a tetra­chloro­zinc(II) salt (CSD refcode COKXAC; Tapia-Benavides *et al.*, 2008 ▸), the solvent-free dichloride salt (NEPKOK; Malecki, 2011 ▸) and the triclinic polymorph of the title compound (NINWIT; Sen *et al.*, 2018 ▸).

**Figure 1:**
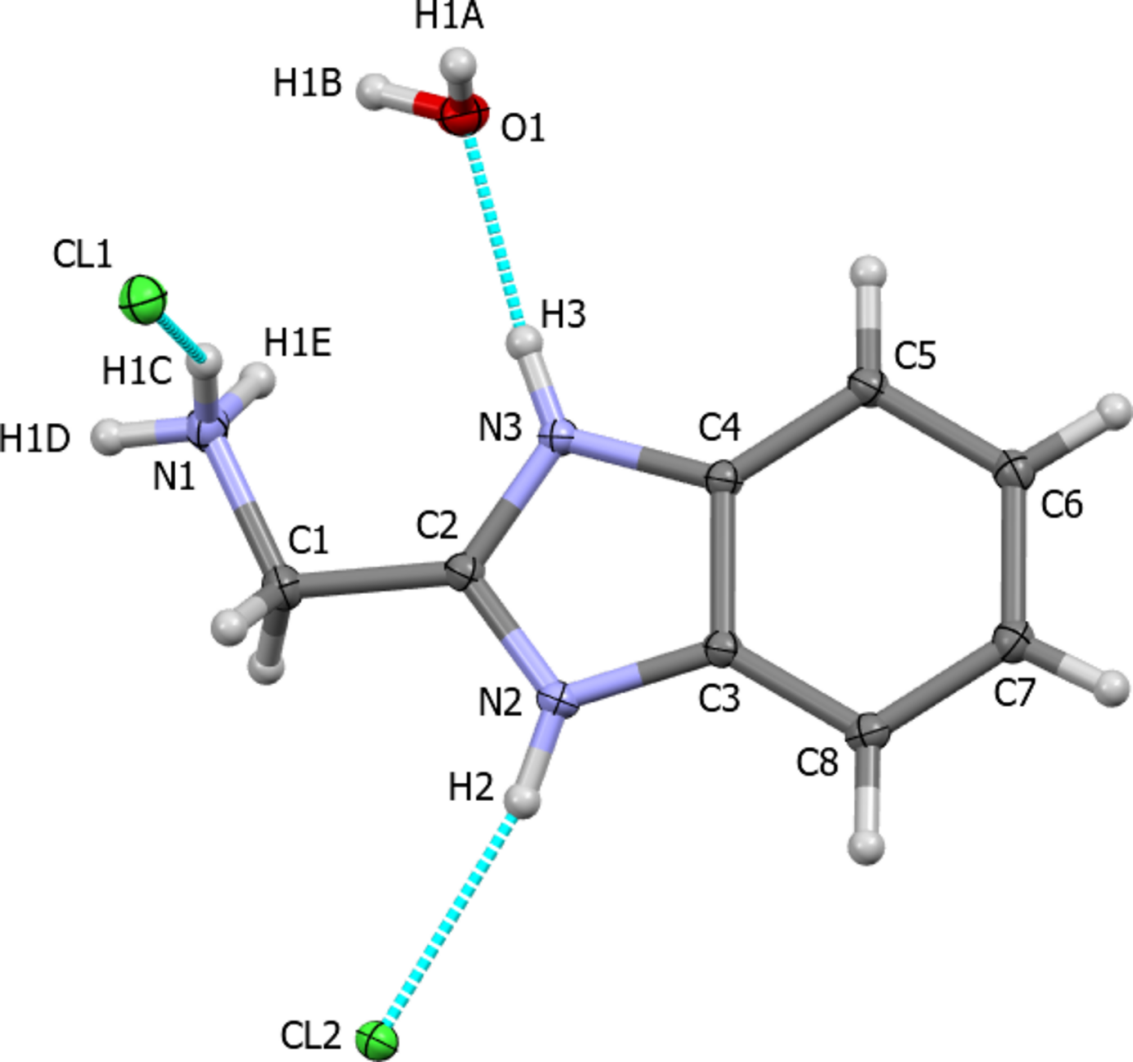
The asymmetric unit of the title compound with 50% probability ellipsoids showing hydrogen bonds as dashed lines.

The title compound crystallizes in a new monoclinic form in space group *P*2_1_/*c* compared with the previously reported triclinic form, in space group *P*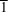 (Sen *et al.*, 2018 ▸). In the monoclinic polymorph, the asymmetric unit contains a benzimidazolium dication, two chloride ions, and a water mol­ecule with *Z*′ = 1 (Fig. 1 ▸). In the more complex triclinic polymorph, the asymmetric unit contains three benzimidazolium dications, six chloride ions and three water mol­ecules with *Z*′ = 3 (Sen *et al.*, 2018 ▸). One notable feature is that in the solvent-free salt (Malecki, 2011 ▸), the pendant CH_2_NH_3_ moiety has a substantial torsion angle of *ca*. 59° relative to the plane of the fused rings, while in both monohydrate polymorphs, in all the unique mol­ecules, that angle is < 10°, so that moiety is close to co-planar with the fused rings.

**Figure 2:**
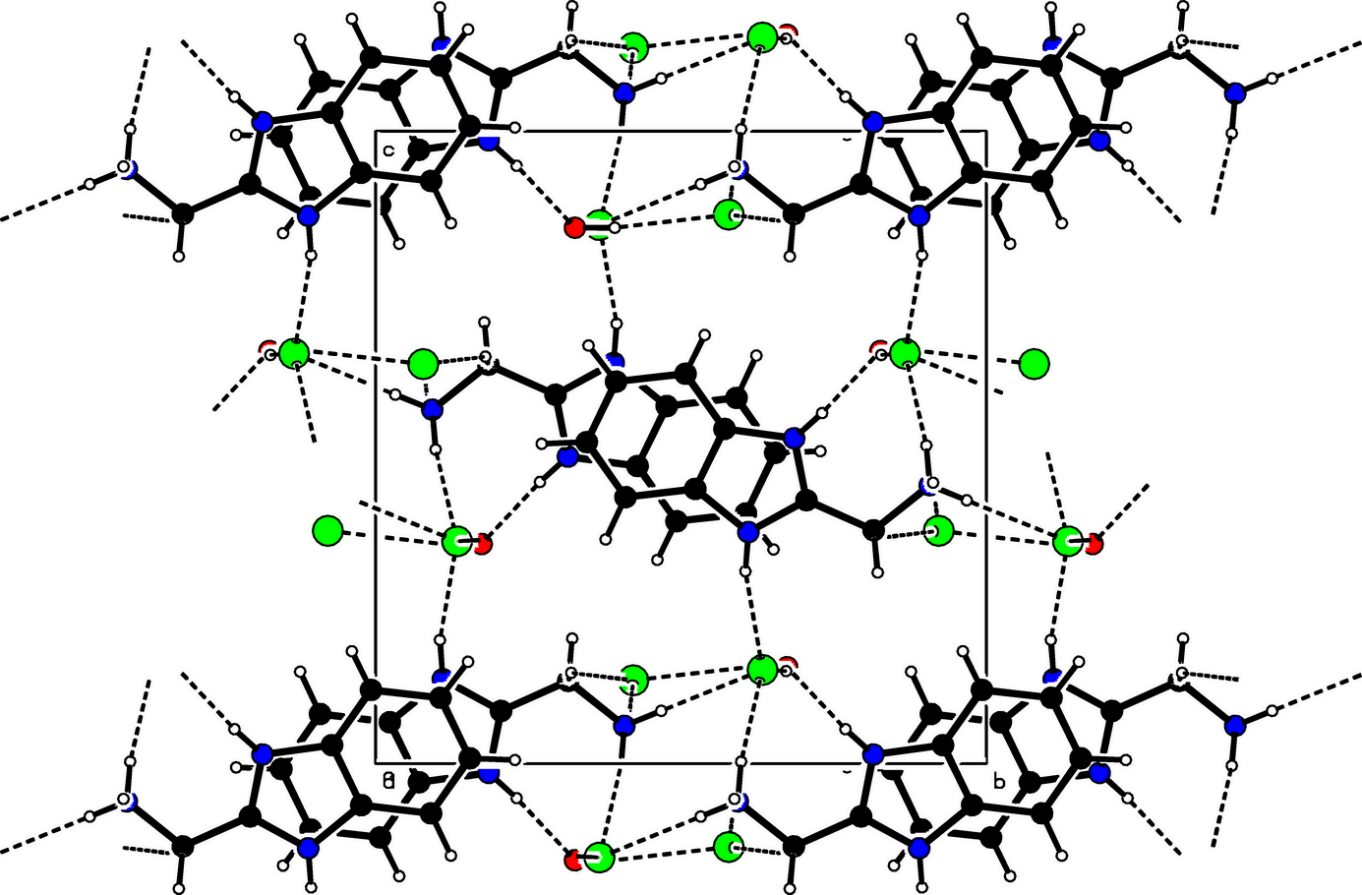
Crystal packing of the title compound viewed down [100].

In the monoclinic polymorph described here, all the N—H groups form strong, charge-assisted, hydrogen bonds to either chloride anions or the water oxygen atom (Table 1 ▸). The water mol­ecule forms O—H⋯Cl links to two chloride ions. The mol­ecules pack in sheets in the *ab* plane and these sheets are then hydrogen bonded to their neighbours, generating a three-dimensional network (Fig. 2 ▸), similar to that of the triclinic polymorph. The cations also display aromatic π–π stacking in the *a*-axis direction with alternate mol­ecules anti-parallel, with a shortest centroid–centroid separation of 3.4071 (4) Å.

## Synthesis and crystallization

The benzimidazolium cation was prepared following the reported procedure (Cescon & Day, 1962 ▸). About 1 mmol (5.46 g) of *o*-phenyl­enedi­amine and 1 mmol (5.68 g) of glycine were mixed and dissolved in 100 ml of hydro­chloric acid (5 mol l^−1^). The solution was refluxed for three days. The reaction mixture was cooled and placed in an ice bath overnight. The resulting purple crystals were isolated from the hydro­chloric acid by filtration, and then recrystallized from ethanol solution, m.p. = 122 °C. From the same re-crystallization, some red crystals of the well known compound *o*-phenyl­enedi­amine di­hydro­chloride [CSD: PHNDMO; Stålhandske, 1974 ▸) were also identified. We have re-determined that structure to a slightly higher precision (Baskaran *et al.*, 2025 ▸)

## Refinement

Crystal data, data collection, and structure refinement details are summarized in Table 2 ▸.

## Supplementary Material

Crystal structure: contains datablock(s) global, I. DOI: 10.1107/S2414314625008685/hb4537sup1.cif

Structure factors: contains datablock(s) I. DOI: 10.1107/S2414314625008685/hb4537Isup2.hkl

Supporting information file. DOI: 10.1107/S2414314625008685/hb4537Isup3.cml

CCDC reference: 2485331

Additional supporting information:  crystallographic information; 3D view; checkCIF report

## Figures and Tables

**Table 1:** Hydrogen-bond geometry (Å, °)

*D*—H⋯*A*	*D*—H	H⋯*A*	*D*⋯*A*	*D*—H⋯*A*
N1—H1*C*⋯Cl1	0.84 (1)	2.32 (1)	3.0898 (8)	152 (1)
N1—H1*D*⋯Cl2^i^	0.83 (1)	2.30 (1)	3.1262 (7)	177 (1)
N1—H1*E*⋯Cl2^ii^	0.86 (1)	2.41 (1)	3.2430 (7)	163 (1)
N2—H2⋯Cl2	0.866 (16)	2.259 (16)	3.1099 (6)	167.2 (14)
N3—H3⋯O1	0.812 (15)	1.911 (16)	2.7197 (9)	173.6 (15)
O1—H1*A*⋯Cl2^iii^	0.80 (1)	2.59 (1)	3.3752 (7)	169 (2)
O1—H1*B*⋯Cl1^iv^	0.81 (1)	2.31 (1)	3.1209 (7)	173 (2)
C1—H1*F*⋯Cl1^v^	0.988 (13)	2.793 (13)	3.7079 (8)	154.2 (10)
C5—H5⋯Cl1^ii^	0.926 (13)	2.960 (13)	3.8515 (7)	162.1 (10)

Symmetry codes: (i) 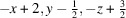; (ii) 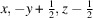; (iii) 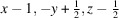; (iv) 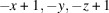; (v) 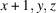.

**Table 2:** Experimental details

Crystal data	
Chemical formula	C_8_H_11_N_3_^2+^·2Cl^−^·H_2_O
*M*_r_	238.11
Crystal system, space group	Monoclinic, *P*2_1_/*c*
Temperature (K)	100
*a*, *b*, *c* (Å)	6.81630 (11), 12.09585 (19), 12.5226 (2)
β (°)	90.7201 (14)
*V* (Å^3^)	1032.39 (3)
*Z*	4
Radiation type	Mo *K*α
μ (mm^−1^)	0.60
Crystal size (mm)	0.18 × 0.15 × 0.07

Data collection	
Diffractometer	Rigaku FRE+ diffractometer with HF Varimax confocal mirrors, a UG2 goniometer and HyPix 6000HE detector
Absorption correction	Analytical (CrystalisPro; Rigaku OD, 2024 ▸)
*T*_min_, *T*_max_	0.988, 0.994
No. of measured, independent and observed [*I* > 2σ(*I*)] reflections	49678, 5003, 4463
*R*_int_	0.057
(sin θ/λ)_max_ (Å^−1^)	0.833

Refinement	
*R*[*F*^2^ > 2σ(*F*^2^)], *wR*(*F*^2^), *S*	0.029, 0.080, 1.06
No. of reflections	5003
No. of parameters	172
No. of restraints	5
H-atom treatment	Only H-atom coordinates refined
Δρ_max_, Δρ_min_ (e Å^−3^)	0.72, −0.22

Computer programs: *CrysAlis PRO* (Rigaku OD, 2024 ▸), *SHELXT2018/2* (Sheldrick, 2015*a* ▸), *SHELXL2019/3* (Sheldrick, 2015*b* ▸) and *SHELXTL* (Sheldrick, 2008 ▸).

## full crystallographic data

### 2-Azaniumylmethyl-1H-benzimidazol-3-ium dichloride monohydrate Crystal data

**Table:**

C_8_H_11_N_3_^2+^·2Cl^−^·H_2_O	*F*(000) = 496
*M**_r_* = 238.11	*D*_x_ = 1.532 Mg m^−^^3^
Monoclinic, *P*2_1_/*c*	Mo *K*α radiation, λ = 0.71073 Å
*a* = 6.81630 (11) Å	Cell parameters from 29865 reflections
*b* = 12.09585 (19) Å	θ = 2.3–38.0°
*c* = 12.5226 (2) Å	µ = 0.60 mm^−^^1^
β = 90.7201 (14)°	*T* = 100 K
*V* = 1032.39 (3) Å^3^	Block, colourless
*Z* = 4	0.18 × 0.15 × 0.07 mm

### 2-Azaniumylmethyl-1H-benzimidazol-3-ium dichloride monohydrate Data collection

**Table:**

Rigaku FRE+ diffractometer with HF Varimax confocal mirrors, a UG2 goniometer and HyPix 6000HE detector	5003 independent reflections
Radiation source: Rotating Anode	4463 reflections with *I* > 2σ(*I*)
Detector resolution: 10 pixels mm^-1^	*R*_int_ = 0.057
profile data from ω–scans	θ_max_ = 36.3°, θ_min_ = 2.3°
Absorption correction: analytical (CrystalisPro; Rigaku OD, 2024)	*h* = −11→11
*T*_min_ = 0.988, *T*_max_ = 0.994	*k* = −20→20
49678 measured reflections	*l* = −20→20

### 2-Azaniumylmethyl-1H-benzimidazol-3-ium dichloride monohydrate Refinement

**Table:**

Refinement on *F*^2^	Primary atom site location: iterative
Least-squares matrix: full	Hydrogen site location: difference Fourier map
*R*[*F*^2^ > 2σ(*F*^2^)] = 0.029	Only H-atom coordinates refined
*wR*(*F*^2^) = 0.080	*w* = 1/[σ^2^(*F*_o_^2^) + (0.0466*P*)^2^ + 0.1488*P*] where *P* = (*F*_o_^2^ + 2*F*_c_^2^)/3
*S* = 1.06	(Δ/σ)_max_ = 0.002
5003 reflections	Δρ_max_ = 0.72 e Å^−^^3^
172 parameters	Δρ_min_ = −0.22 e Å^−^^3^
5 restraints	

### 2-Azaniumylmethyl-1H-benzimidazol-3-ium dichloride monohydrate Special details

**Table:**

Geometry. All esds (except the esd in the dihedral angle between two l.s. planes) are estimated using the full covariance matrix. The cell esds are taken into account individually in the estimation of esds in distances, angles and torsion angles; correlations between esds in cell parameters are only used when they are defined by crystal symmetry. An approximate (isotropic) treatment of cell esds is used for estimating esds involving l.s. planes.
Refinement. The H atoms atoms were located in a difference Fourier map and their coordinates allowed to refine freely. *U*_iso_(H) values were also freely refined except for those on N1 and C5–C8, which were contstrained and tied to those of the carrier atom with *U*_iso_(H) = 1.5 *U_eq_*(N) and 1.2 *U_eq_*(C).

### 2-Azaniumylmethyl-1H-benzimidazol-3-ium dichloride monohydrate Fractional atomic coordinates and isotropic or equivalent isotropic displacement parameters (Å^2^)

**Table:**

	*x*	*y*	*z*	*U*_iso_*/*U*_eq_	
N1	0.78913 (11)	0.09281 (6)	0.55978 (6)	0.01490 (11)	
H1C	0.6658 (17)	0.0874 (11)	0.5557 (11)	0.022*	
H1D	0.824 (2)	0.0323 (10)	0.5836 (11)	0.022*	
H1E	0.839 (2)	0.0984 (11)	0.4968 (9)	0.022*	
C1	0.84801 (12)	0.18467 (7)	0.63089 (6)	0.01495 (13)	
H1F	0.991 (2)	0.1798 (11)	0.6436 (10)	0.019 (3)*	
H1G	0.777 (2)	0.1781 (11)	0.6978 (11)	0.023 (3)*	
C2	0.80152 (10)	0.29464 (6)	0.58338 (6)	0.01152 (11)	
N2	0.83825 (10)	0.38973 (5)	0.63375 (5)	0.01193 (11)	
H2	0.896 (2)	0.3946 (13)	0.6957 (13)	0.030 (4)*	
C3	0.79320 (10)	0.47665 (6)	0.56549 (6)	0.01090 (11)	
C4	0.72336 (10)	0.42885 (6)	0.47064 (6)	0.01071 (11)	
N3	0.73024 (9)	0.31486 (5)	0.48549 (5)	0.01132 (10)	
H3	0.682 (2)	0.2691 (12)	0.4455 (12)	0.026 (3)*	
C5	0.66377 (11)	0.49262 (6)	0.38340 (6)	0.01216 (12)	
H5	0.6100 (18)	0.4613 (10)	0.3222 (10)	0.015*	
C6	0.67891 (11)	0.60627 (6)	0.39581 (6)	0.01288 (12)	
H6	0.6386 (19)	0.6514 (11)	0.3388 (10)	0.015*	
C7	0.74867 (11)	0.65425 (6)	0.49147 (6)	0.01293 (12)	
H7	0.7560 (19)	0.7282 (11)	0.4942 (10)	0.016*	
C8	0.80654 (11)	0.59081 (6)	0.57854 (6)	0.01269 (12)	
H8	0.8465 (19)	0.6234 (11)	0.6454 (10)	0.015*	
O1	0.54805 (9)	0.17363 (5)	0.34741 (5)	0.01701 (11)	
H1A	0.431 (2)	0.1721 (15)	0.3536 (14)	0.043 (4)*	
H1B	0.577 (2)	0.1084 (12)	0.3475 (13)	0.038 (4)*	
Cl1	0.35804 (3)	0.07804 (2)	0.63181 (2)	0.01669 (5)	
Cl2	1.05821 (3)	0.36655 (2)	0.85168 (2)	0.01277 (5)	

### 2-Azaniumylmethyl-1H-benzimidazol-3-ium dichloride monohydrate Atomic displacement parameters (Å^2^)

**Table:**

	*U*^11^	*U*^22^	*U*^33^	*U*^12^	*U*^13^	*U*^23^
N1	0.0179 (3)	0.0114 (3)	0.0153 (3)	0.0012 (2)	0.0001 (2)	0.0004 (2)
C1	0.0182 (3)	0.0123 (3)	0.0142 (3)	−0.0003 (2)	−0.0031 (2)	0.0022 (2)
C2	0.0113 (3)	0.0122 (3)	0.0110 (3)	−0.0004 (2)	−0.0012 (2)	0.0006 (2)
N2	0.0129 (2)	0.0126 (3)	0.0102 (2)	−0.00059 (19)	−0.00245 (19)	0.00026 (19)
C3	0.0108 (3)	0.0118 (3)	0.0101 (3)	−0.0005 (2)	−0.00105 (19)	−0.0002 (2)
C4	0.0107 (3)	0.0110 (3)	0.0104 (3)	0.0001 (2)	−0.0005 (2)	−0.0004 (2)
N3	0.0121 (2)	0.0108 (2)	0.0109 (2)	−0.00018 (19)	−0.00197 (18)	−0.00061 (19)
C5	0.0126 (3)	0.0134 (3)	0.0104 (3)	0.0005 (2)	−0.0013 (2)	0.0000 (2)
C6	0.0131 (3)	0.0131 (3)	0.0124 (3)	0.0003 (2)	−0.0001 (2)	0.0015 (2)
C7	0.0128 (3)	0.0118 (3)	0.0142 (3)	−0.0007 (2)	0.0003 (2)	−0.0001 (2)
C8	0.0127 (3)	0.0127 (3)	0.0126 (3)	−0.0016 (2)	−0.0008 (2)	−0.0017 (2)
O1	0.0162 (3)	0.0157 (3)	0.0190 (3)	0.00068 (19)	−0.0038 (2)	−0.0036 (2)
Cl1	0.01671 (9)	0.01516 (9)	0.01815 (9)	0.00088 (6)	−0.00154 (6)	−0.00008 (6)
Cl2	0.01447 (8)	0.01281 (8)	0.01098 (7)	0.00039 (5)	−0.00244 (5)	−0.00001 (5)

### 2-Azaniumylmethyl-1H-benzimidazol-3-ium dichloride monohydrate Geometric parameters (Å, º)

**Table:**

N1—C1	1.4763 (10)	C4—N3	1.3920 (9)
N1—H1C	0.844 (12)	C4—C5	1.3938 (10)
N1—H1D	0.825 (11)	N3—H3	0.812 (15)
N1—H1E	0.864 (11)	C5—C6	1.3872 (11)
C1—C2	1.4896 (11)	C5—H5	0.926 (13)
C1—H1F	0.988 (13)	C6—C7	1.4085 (11)
C1—H1G	0.975 (14)	C6—H6	0.937 (13)
C2—N2	1.3340 (9)	C7—C8	1.3865 (10)
C2—N3	1.3355 (9)	C7—H7	0.897 (13)
N2—C3	1.3871 (9)	C8—H8	0.961 (13)
N2—H2	0.866 (16)	O1—H1A	0.802 (14)
C3—C8	1.3933 (10)	O1—H1B	0.813 (14)
C3—C4	1.3991 (10)		
			
C1—N1—H1C	111.0 (9)	C8—C3—C4	122.02 (7)
C1—N1—H1D	111.9 (10)	N3—C4—C5	131.45 (6)
H1C—N1—H1D	103.9 (13)	N3—C4—C3	106.58 (6)
C1—N1—H1E	112.7 (9)	C5—C4—C3	121.96 (6)
H1C—N1—H1E	110.5 (13)	C2—N3—C4	108.38 (6)
H1D—N1—H1E	106.5 (13)	C2—N3—H3	125.4 (11)
N1—C1—C2	112.11 (6)	C4—N3—H3	125.4 (11)
N1—C1—H1F	108.2 (8)	C6—C5—C4	116.11 (7)
C2—C1—H1F	108.8 (8)	C6—C5—H5	121.8 (8)
N1—C1—H1G	108.9 (8)	C4—C5—H5	122.0 (8)
C2—C1—H1G	108.1 (8)	C5—C6—C7	121.83 (7)
H1F—C1—H1G	110.7 (11)	C5—C6—H6	118.1 (8)
N2—C2—N3	109.88 (6)	C7—C6—H6	120.1 (8)
N2—C2—C1	122.91 (6)	C8—C7—C6	122.04 (7)
N3—C2—C1	127.10 (6)	C8—C7—H7	120.5 (8)
C2—N2—C3	108.87 (6)	C6—C7—H7	117.5 (8)
C2—N2—H2	124.2 (11)	C7—C8—C3	116.02 (6)
C3—N2—H2	126.5 (11)	C7—C8—H8	122.2 (8)
N2—C3—C8	131.71 (7)	C3—C8—H8	121.7 (8)
N2—C3—C4	106.27 (6)	H1A—O1—H1B	102.8 (17)
			
N1—C1—C2—N2	178.82 (7)	C1—C2—N3—C4	−175.26 (7)
N1—C1—C2—N3	−5.29 (11)	C5—C4—N3—C2	179.19 (8)
N3—C2—N2—C3	−1.28 (8)	C3—C4—N3—C2	−0.44 (8)
C1—C2—N2—C3	175.23 (7)	N3—C4—C5—C6	−179.18 (7)
C2—N2—C3—C8	−179.35 (8)	C3—C4—C5—C6	0.40 (11)
C2—N2—C3—C4	0.97 (8)	C4—C5—C6—C7	−0.60 (11)
N2—C3—C4—N3	−0.32 (8)	C5—C6—C7—C8	0.14 (12)
C8—C3—C4—N3	179.96 (7)	C6—C7—C8—C3	0.54 (11)
N2—C3—C4—C5	−179.99 (7)	N2—C3—C8—C7	179.61 (7)
C8—C3—C4—C5	0.28 (11)	C4—C3—C8—C7	−0.74 (11)
N2—C2—N3—C4	1.07 (8)		

### 2-Azaniumylmethyl-1H-benzimidazol-3-ium dichloride monohydrate Hydrogen-bond geometry (Å, º)

**Table:**

*D*—H···*A*	*D*—H	H···*A*	*D*···*A*	*D*—H···*A*
N1—H1*C*···Cl1	0.84 (1)	2.32 (1)	3.0898 (8)	152 (1)
N1—H1*D*···Cl2^i^	0.83 (1)	2.30 (1)	3.1262 (7)	177 (1)
N1—H1*E*···Cl2^ii^	0.86 (1)	2.41 (1)	3.2430 (7)	163 (1)
N2—H2···Cl2	0.866 (16)	2.259 (16)	3.1099 (6)	167.2 (14)
N3—H3···O1	0.812 (15)	1.911 (16)	2.7197 (9)	173.6 (15)
O1—H1*A*···Cl2^iii^	0.80 (1)	2.59 (1)	3.3752 (7)	169 (2)
O1—H1*B*···Cl1^iv^	0.81 (1)	2.31 (1)	3.1209 (7)	173 (2)
C1—H1*F*···Cl1^v^	0.988 (13)	2.793 (13)	3.7079 (8)	154.2 (10)
C5—H5···Cl1^ii^	0.926 (13)	2.960 (13)	3.8515 (7)	162.1 (10)

Symmetry codes: (i) −*x*+2, *y*−1/2, −*z*+3/2; (ii) *x*, −*y*+1/2, *z*−1/2; (iii) *x*−1, −*y*+1/2, *z*−1/2; (iv) −*x*+1, −*y*, −*z*+1; (v) *x*+1, *y*, *z*.
